# Cooperative and Competitive Contextual Effects on Social Cognitive and Empathic Neural Responses

**DOI:** 10.3389/fnhum.2018.00218

**Published:** 2018-06-11

**Authors:** Minhye Lee, Hyun Seon Ahn, Soon Koo Kwon, Sung-il Kim

**Affiliations:** ^1^Department of Education, Brain and Motivation Research Institute (bMRI), Korea University, Seoul, South Korea; ^2^Center for Teaching, Learning, and Technology, Inha University, Incheon, South Korea; ^3^Education Performance Evaluation Center, Dankook University, Yongin, South Korea

**Keywords:** cooperation, competition, helping, interfering, empathy, ventromedial prefrontal cortex (vmPFC), anterior insular cortex (AIC), dorsal anterior cingulate cortex (dACC)

## Abstract

We aimed to differentiate the neural responses to cooperative and competitive contexts, which are the two of the most important social contexts in human society. Healthy male college students were asked to complete a Tetris-like task requiring mental rotation skills under individual, cooperative, and competitive contexts in an fMRI scanner. While the participants completed the task, pictures of others experiencing pain evoking emotional empathy randomly appeared to capture contextual effects on empathic neural responses. Behavioral results indicated that, in the presence of cooperation, participants solved the tasks more accurately and quickly than what they did when in the presence of competition. The fMRI results revealed activations in the dorsolateral prefrontal cortex (dlPFC) and dorsomedial prefrontal cortex (dmPFC) related to executive functions and theory of mind when participants performed the task under both cooperative and competitive contexts, whereas no activation of such areas was observed in the individual context. Cooperation condition exhibited stronger neural responses in the ventromedial prefrontal cortex (vmPFC) and dmPFC than competition condition. Competition condition, however, showed marginal neural responses in the cerebellum and anterior insular cortex (AIC). The two social contexts involved stronger empathic neural responses to other’s pain than the individual context, but no substantial differences between cooperation and competition were present. Regions of interest analyses revealed that individual’s trait empathy modulated the neural activity in the state empathy network, the AIC, and the dorsal anterior cingulate cortex (dACC) depending on the social context. These results suggest that cooperation improves task performance and activates neural responses associated with reward and mentalizing. Furthermore, the interaction between trait- and state-empathy was explored by correlation analyses between individual’s trait empathy score and changing empathic brain activations along with the exposure to the cooperative and competitive social contexts.

## Introduction

Cooperation and competition are the two of the most important social behaviors in human society. From a traditional evolutionary perspective, cooperation involves the sharing of resources in order to enhance group security and to ensure reliable access to important resources (Trivers, 1971). In contrast, competition involves the monopolization of resources to maximize individual advantages based on the survival of the fittest (Freeman, 1974). A great deal of research has shown that the social interactions associated with cooperation and competition have a lasting effect on human behavior and motivation.

However, there has been long-lasting debate over the relative importance of these two social behaviors (for a review, see Murayama and Elliot, 2012). Adherents to a humanistic perspective contend that, by leading people toward shared goals, cooperation is more beneficial for human motivation and productivity (e.g., Johnson et al., 1981; Deci and Ryan, 1985; Kohn, 1986). By cooperating, individuals can complement each other and realize the associated benefits in the pursuit of a shared goal. In contrast, behaviorists claim that competition promotes productivity by creating fierce rivalry between individuals (e.g., Festinger, 1954; Locke, 1968; Michaels, 1977). In the face of competition, individuals tend to maximize their potential to overcome their opponents.

This ongoing debate has now moved onto more fundamental questions such as why and how cooperation and competition result in different consequences despite their shared feature, social interaction among human beings. One widely used methodology for determining the underlying mechanisms of human behavior is the neuroscientific approach. This method can shed new light on underrepresented features of cooperation and competition beyond observable behavioral and performance outcomes. In line with this idea, we expect that an investigation of the neural responses to cooperation and competition could increase the understanding of their social cognitive and emotional aspects. In the remainder of this section, we will first summarize existing neuroscientific literature on cooperation and competition and then highlight unresolved issues that will be addressed in the current study.

Several neuroscientists have attempted to elucidate the different neural responses to cooperation and competition by exploring social cognitive processes in the human brain (Adolphs, 2003; Lieberman, 2007). Game theory has been widely adopted to explain dynamic changes in decision-making in different contexts, including cooperation and competition. For instance, game theorists have used the dictator game, ultimatum game, trust game, and the prisoner’s dilemma game to investigate changes of decision-making processes depending on the opponent’s thoughts and choices (for a review, see Rilling and Sanfey, 2011). In this paradigm, cooperation is characterized by reciprocal, fair, and altruistic cooperators, whereas competition is created through the introduction of un-reciprocal, unfair, and selfish deceivers.

It is clear from previous research that both cooperation and competition activate social-cognitive neural responses related to the reading of others’ minds and the prediction of others’ future behavior, a process known as “mentalizing.” What extent to which neural mechanism is interconnected with mentalizing network during cooperation and competition, respectively, can explain differences between the two social behaviors. Cooperation activates reward systems such as the ventromedial prefrontal cortex (vmPFC) and striatum, and mentalizing regions such as the dorsomedial prefrontal cortex (dmPFC), temporoparietal junction (TPJ), and superior temporal sulcus (STS; McCabe et al., 2001; Rilling et al., 2002; Decety et al., 2004; King-Casas et al., 2005; Elliott et al., 2006). In contrast, competition activates inference-related brain regions such as the inferior frontal gyrus (IFG) and dorsolateral prefrontal cortex (dlPFC) and the mentalizing regions (Decety et al., 2004; Lissek et al., 2008; Halko et al., 2009). These findings lend support to the view that, from an evolutionary perspective, humans are cooperative and altruistic creatures as it activates reward circuits and adaptive social cognitive networks. However, competition appears to make individuals engaged in reading and monitoring opponents’ intention during the game as a means of betrayal or deception.

Cooperation and competition can lead to distinct emotional neural responses to other people, such as empathy since they are inevitably accompanied by emotional reactions to or judgment of other people (Gonzalez-Liencres et al., 2013). Unfortunately, few studies have focused on differential empathic responses to others’ emotions depending on the social contexts (Akitsuki and Decety, 2009; Hein et al., 2010). Singer et al. (2006) found that, when participants observed the pain of an unfair confederate, there was reduced activation of empathy-related brain regions, including the dorsal anterior cingulate cortex (dACC) and the anterior insular cortex (AIC) compared to when they witnessed the pain of a fair confederate. In a similar vein, people are better able to empathize with the pain of in-group members and members of the same race than that of out-group members and other races (Xu et al., 2009; de Dreu, 2010; Hein et al., 2010; Masten et al., 2010).

These differences in the emotional response to other individuals can be understood in relation to the nature of cooperation and competition. Cooperation provides the participants with shared goals and encourages them to equate themselves with each other. In this sense, fair confederates, in-group members, and “same race” individuals are assumed to be on the same side and trustworthy. In contrast, competition assumes that other individuals will have opposing or conflicting goals, thus encouraging participants to separate or distinguish themselves from others. Unfair confederates, out-group members, and “other race” individuals therefore generate feelings of wariness, psychological distance, and uncertainty about their trustworthiness. Based on this, we can assume that cooperation would lead to more empathic responses toward others’ pain, whereas competition would not.

Individual differences in sensitivity toward external social and emotional signals are likely to modulate the social contextual effects on human brain’s social cognitive and emotional responses, which in turn would lead to subsequent behavioral changes (Masten et al., 2011). For a representative personality trait interacting with social environments, trait empathy can play an essential role as a modulator of state empathy under different social contexts. By definition, trait empathy refers to an individual difference in the appraisal of and responsiveness to other people’s emotional experiences (Mehrabian and Epstein, 1972; Chlopan et al., 1985). Being aware of or sensitive to the emotions of others increases prosocial behavior such as cooperation, sharing, and helping (Batson et al., 1995; Batson and Ahmad, 2001; Rumble et al., 2010). In addition, neuroscientists have found positive correlations between trait empathy and state empathic brain activations when individuals observe the pain of others (Singer et al., 2004, 2006; Jabbi et al., 2007; Saarela et al., 2007). Specifically, more empathic individuals tend to exhibit greater activations in the AIC and dACC, which are known to be part of the state empathy network, as well as cognitive mentalizing regions, such as the dmPFC, TPJ, temporal pole, and precuneus (Masten et al., 2011).

Extending these findings, empathic individuals are expected to demonstrate stronger brain activations in the empathy and mentalizing networks particularly when helping other people under a cooperative context. They might perceive the cooperative context as being consistent with their trait-like motivation for prosocial behaviors and perspective taking. Thereby, this type of individuals could be more readily immersed in a task under the cooperative context than under the other social contexts raising hostility or competition. In this sense, they may not be able to completely concentrate on the task in a competitive context because beating or disrupting the other person in a game-like task is perceived to be incongruent with their trait. Consequently, they are likely to have weaker activation in the brain regions related to state empathy and mentalizing when they observe the pain of others during competition. Thus, we aim to examine the interactions between trait- and state- empathy under differential social contexts.

Despite the above-mentioned efforts to understand the fundamental mechanisms underlying cooperation and competition in particular regarding their social cognitive and emotional influences, there remain a number of issues to be addressed. First, the potential confounding effect of feedback hinders the better understanding of the core mechanisms of cooperation and competition. Success/failure feedback moderates the influence of cooperative and competitive social contexts on participants’ cognitive and motivational processes (Ames and Felker, 1979; Ames, 1981, 1984). This is particularly important when monetary rewards are used as an outcome of social interaction. It has been reported that cooperation boosts reward-related brain activation in the vmPFC particularly when feedback of cooperation is provided in monetary form (Elliott et al., 2006). It seems that cooperative behavior was required in this study for the participants to receive a reward. It is hard to distinguish neural mechanisms of cooperation from reward processing activated by the financial incentive, as a reward of cooperation. In other words, there is a possibility that the results of the vmPFC activation were confounded by the effects of the financial incentives as feedback. Hence, it is not yet clear whether the neural observations would be the same without the presence of feedback. In order to investigate purely contextual effects on human psychological processes, we need to control feedback information related to social incentives.

Second, past neuroscientific studies have disregarded the effects of contextual cues related to cooperation and competition on motivational and psychological processes. Ames and Felker (1979) found that the nature of reading material prompted children to make different evaluations of the performance outcomes for main characters in socially contextualized stories. For instance, children who read a story about competition were more likely to attribute the main character’s success to a strong inborn ability in comparison to others who read a story related to cooperation. In other words, an individual’s attitude toward performance can be influenced by simple contextual stimuli in the absence of actual interpersonal supportive transactions. This limitation can be overcome by narrowing the definition of cooperation and competition to a simple contextual cue by focusing on the simplified behavioral goals: helping or disrupting others to maximize one’s own benefits (Decety et al., 2004).

Third, most existing literature dealing with the effect of cooperation and competition has focused on cognitive aspects, such as brain activation during a cognitively demanding task or complex decision making (Rilling et al., 2002; Decety et al., 2004; Elliott et al., 2006; Halko et al., 2009). However, cooperative and competitive contexts could influence emotional processes in different ways, particularly toward other people due to social features of cooperation and competition. By exploring the effects of cooperation and competition on the more emotional and affective aspects, we would extend our understanding of underlying mechanisms of cooperation and competition.

Finally, there is a need to explore the potential interaction between trait- and state-empathy under differential social contexts. People with different personality traits interpret and determine their reactions toward external events in different ways, particularly when dealing with social interactions with others. Trait empathy, which is the sensitivity to other people’s emotions, would moderate an individual’s appraisal of a social context (Bernhardt and Singer, 2012).

Therefore, in the present study, we primarily aimed to investigate the effects of cooperative and competitive social contexts on psychological processes while deliberately excluding potential confounding effects of reciprocal social interaction among participants and those of monetary feedback. We also aimed to explore the modulation effect of trait empathy on state empathy in cooperative and competitive social contexts. Throughout the review of literature, we generated three research questions and formulated hypotheses along with them as follows.

Research question 1: Would cooperative and competitive contexts have distinctive impacts on social cognitive neural responses while individuals solve spatial perception task from other’s perspective?

According to the previous research using the space perception task (Decety et al., 2004), under both cooperative and competitive contexts, common neural mechanism was found in the brain activation of inferior parietal lobe (IPL), superior frontal gyrus (SFG), and superior parietal lobe (SPL), which are associated with task engagement in spatial perception task and mentalizing. In addition to these commonly activated regions, the cooperation condition more strongly activated theory of mind network (i.e., dmPFC, precuneus) and reward network (i.e., vmPFC) than the competition condition. To extend these findings, we could draw hypotheses that under both cooperative and competitive contexts, participants would demonstrate better performances in the spatial task compared to the individually performing context. Thereby, they would show activations related to the spatial cognitive processing in the IPL including angular gyrus (AG) and supramarginal gyrus (Anderson, 1987) and the dlPFC (Ptak et al., 2017) compared to when they solve the task independently without any social cues. About the distinctive network between the two social contexts, cooperation would lead to the stronger recruitment of mentalizing regions, such as the dmPFC and TPJ than competition would do (Saxe and Kanwisher, 2003; Saxe, 2006). Competition would lead to activation in the self-other distinction and self-centered network, such as the precuneus (Cavanna and Trimble, 2006). Furthermore, in a cooperative context, participants would exhibit activation in the reward circuit including the vmPFC and striatum because the act of helping and sharing goals with others would lead to emotional satisfaction (Decety et al., 2004; Elliott et al., 2006). In contrast, in a competitive context, they would be likely to show activation in regions associated with negative emotions, such as the amygdala and AIC, because interfering and deceiving others would cause emotional distress and negative arousal (Decety et al., 2004).

Research question 2: Would cooperative and competitive social contexts lead to differential neural activations in empathic network?

Our brain’s empathy network sensitively responds to external social cues defining characteristics of the other people or the environments due to its contagious nature. More precisely, people tend to empathize with the pain of other people who are psychologically close to themselves, such as a spouse, boy/girlfriend, or in-group members (de Dreu, 2010; Hein et al., 2010; Masten et al., 2010). Otherwise, environmental cues of psychological distance from oneself and the other people result in a different degree of state empathy (Singer et al., 2006; Akitsuki and Decety, 2009). From this perspective, cooperation can make people narrow the psychological distance from the other person by sharing the goal, whereas competition would make people distant and indifferent to each other by disrupting the other’s goal. To synthesize, individuals would become more sensitive to pain stimuli in the presence of cooperation, leading to activation in pain-related empathy brain regions, such as the AIC, dACC, and the pre-supplementary motor area (pre-SMA) (Jackson et al., 2005; Singer et al., 2006). In the face of competition, however, their empathy network was likely to be suppressed to attain their goal of winning the game by beating competitors.

Research question 3: Would individual’s trait empathy modulate the influences of social contexts on the state empathy?

Trait empathy could function as a significant modulator in the contextual effects on empathic responses and appraisals of other’s emotions at the moment (Mehrabian and Epstein, 1972; Chlopan et al., 1985). Since trait empathy could play an important role in perceiving and interpreting social emotions of other people, the abovementioned empathic neural responses could interact with the level of trait empathy (Saarela et al., 2007). People with high trait empathy showed stronger activations in the empathy network consisting of the AIC and dACC, when they witnessed the victim of social exclusion (Masten et al., 2011). That is, individuals who are more susceptible to other’s emotions tended to empathize with the social pain of the victim of bullying more strongly compared to the others with lower trait empathy score. To connect the previous finding to the current study, there would be an interaction between trait- and state-empathy on the social cognitive and empathic neural responses. More specifically, when participants solve the task under a cooperative context, the more empathic individuals were expected to show stronger activation in the mentalizing- and reward-related brain regions, such as the dmPFC and vmPFC. When they are exposed to empathy-evoked stimuli during cooperation, the more empathic participants would show stronger activation in the empathy related brain regions, such as the AIC and dACC.

## Materials and Methods

### Participants

Sixteen right-handed male undergraduate students (mean age = 23.2 ± 2.3 years) were recruited through a university community website^1^. The sample size was determined by a power analysis with the strong effect size for testing difference between two dependent means using G^∗^Power 3.1.9.2 (Faul et al., 2007). None of the participants had a history of psychiatric or neurological disorders or psychopharmacological treatment. Handedness was evaluated using the Hand Usage Questionnaire (Chapman and Chapman, 1987). This study was carried out in accordance with the recommendations of Institutional Review Board, Korea University, South Korea. This protocol was approved by the Korea University Institutional Review Board. All subjects gave written informed consent in accordance with the Declaration of Helsinki and received a modest payment for participating in the study (30,000 KRW; roughly equivalent to 30 USD).

### Tasks and Procedures

Before entering the scanner, all participants were required to undergo approximately 30 min of instruction. This was conducted in a small room containing a table, four chairs, and a laptop. Participants were introduced to and became acquainted with two confederates (one is a cooperator, the other is a competitor) to allow the cooperative and competitive contexts to be established later. These confederates were male undergraduate students and were about the same mean age as the participants (22.3 years). The role of two confederates was unknown to participants until the actual experiment started in the fMRI machine. All behavioral and personal data for the two actors were excluded from the study because they were purely used to manipulate the social context for the experiment. After being introduced to the confederates, the participants were left alone with a well-trained instructor and PowerPoint-based instructions regarding the experimental procedure, task stimuli, and the use of response buttons in the room.

**FIGURE 1:**
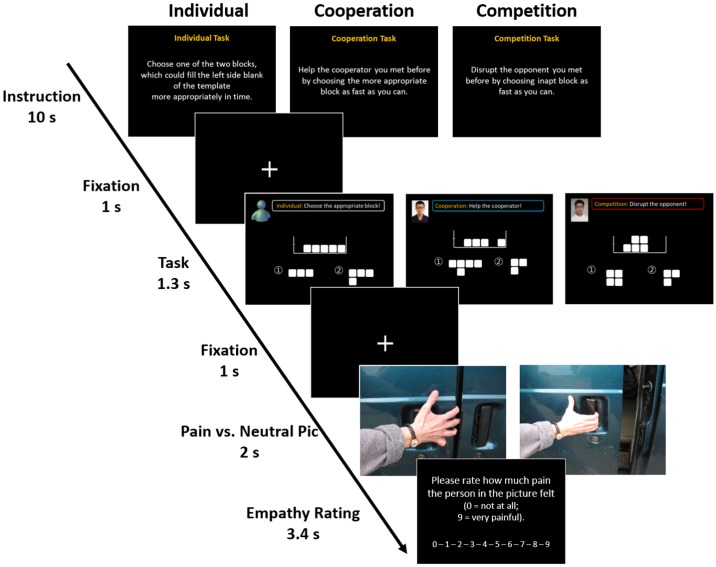
Experimental design. The game consisted of three sessions representing different social contexts: individual, cooperative, and competitive contexts. The task asked the participants to choose between two different blocks according to the instruction for each trial within the allotted time (1.3 s). Each trial was independent of the others, so the participants did not need to remember or memorize their previous trials. In the individual task, participants were instructed to choose one of two blocks which could correctly fill the space on the left side of the template. In the cooperative task, participants were instructed to *help* a cooperator *by choosing the more appropriate block* as fast as they could. In the competitive task, they were asked to *choose the ill-fitting block to interfere with* their opponent’s task performance. While performing the task, 25 empathy-evoking stimuli and 10 neutral stimuli were randomly presented in order to make participants empathize with the person in the picture. Each picture was presented for 2 s and participants were asked to rate how much pain the person in the picture felt on a 10-point Likert scale within 3.4 s.

The instructor initially explained the purpose of the experiment as “an attempt to examine the ability to make instant decisions and, at the same time, an attempt to satisfy the task rules so as to maximize one’s reward” and introduced the Tetris-like game the participants were to play (**Figure 1**). The game was played under three sets of conditions designed to alter the social context and all subjects were instructed to perform three Tetris-like tasks that differed according to their context: individual, cooperative, or competitive. In the game, the participants were asked to choose between two blocks based on the instruction given for each context within the allotted time (1.3 s). Each version of the task was independent of the others, so the participants did not need to memorize their previous trials. In the individual version of the task, the participants were instructed to choose one of two blocks to correctly fill the left side space on the template. In the cooperative version, the participants were instructed to *help* their cooperator *by choosing the most appropriate block* as fast as they could. In the competitive version, they were asked to *choose the ill-fitting block in order to disrupt* their opponent’s task performance. For all three versions of the task, the participants were also told that, depending on their accuracy and response time, they could receive an additional monetary reward of up to $5 each time they completed the task. In other words, the participants were asked to satisfy the rules for each version of the task in order to gain the extra money. The instructor then led participants to believing that they would play the game with confederates in separate locations through a series of trials. Participants were not informed about how much reward their opponents would be paid; rather, they were only informed about the maximum amount of $5 they could get from each condition. A mock test (10 trials for each version of the task, 30 trials in total) was then conducted for 10 min.

After acquainting themselves thoroughly with the instructions and the operation of the apparatus (a 10-button mouse), participants were placed in the scanner. The mixed block/event-related design consisted of three runs of approximately 7 min each for a total of 21 min. To maximize the potency of the social contexts, only one version of the three tasks (i.e., individual, cooperative, and competitive) was performed for each fMRI run. Because each fMRI run consisted of 10 s worth of instruction and 69 independent trials, participants were exposed to 207 trials in total. Following the 10-s screen of instructions that was presented once at the start of each run, each trial was conducted as follows. First, a 1-s fixation cross was presented. This was followed by the task screen. For the cooperative and competitive versions of the task, a picture of the confederate’s face was displayed in the top left corner, next to a shortened version of the instructions. This screen was shown to the participants for 1.3 s. Another 1-s fixation cross screen followed each task trial. While participants were performing the task, one of 25 empathy-evoking images and 10 neutral images randomly appeared in order to make participants empathize with the person in the empathy evoking pictures (Jackson et al., 2005). Because these pictures have been widely used in neuroimaging studies investigating empathy network (Lamm et al., 2007, 2011), we also adopted them with the authors’ permission. Each picture was presented for 2 s and participants were asked to respond how much pain the person in the picture was experiencing using a 10-point Likert scale within 3.4 s. The trials and pictures were presented in a pseudo-randomized order to equalize the sequence and the total number of trials and pictures for each run. The order of the presentation of second and third runs (the cooperative and competitive versions) was counterbalanced across the participants.

Following the fMRI scanning, each participant had a face-to-face interview of approximately 10 min in length. The researchers briefed the participants on the purpose of the experiment and the pseudo-social players (i.e., the confederates). The researchers explained the real purpose of the experiment as “an attempt to investigate the effects of social context on the neural correlates of social behavior.” Furthermore, they explained that the two confederates engaged in the study to manipulate the social contexts of the experiment.

### Questionnaire

Despite the growing number of studies on social interaction, little is known about how neural activation in social contexts differs depending on an individual’s trait empathy levels. Thus, in order to explore the neural correlates for social context and empathy, we assessed the Emotional Empathic Tendency Scale (Mehrabian and Epstein, 1972), which has been validated and translated into Korean (Seol et al., 2006). This scale consists of 33 items and has been used to measure individual differences in the tendency to empathize with others. Each item was rated on a five-point Likert scale ranging from 1 (strongly disagree) to 5 (strongly agree). The Cronbach’s alpha coefficient for this scale was 0.85.

### fMRI Data Acquisition

Functional magnetic resonance imaging was performed using a 3T Phillips Intera Achieva MRI scanner (Philips Medical Systems, Andover, MA, United States) to collect functional images (single-shot gradient echo planar imaging sequence, TR = 2000 ms, TE = 30 ms, flip angle = 90°, sequential ascending order, 36 3-mm-thick axial slices with no gap, FOV = 240 mm). Structural images were acquired after the first experimental run. During the scan, participants completed three runs of the Tetris game. An fMRI head restraint kit (e.g., foam pads and head strap) was used to prevent head motion. The stimuli were presented using a laboratory computer running E-Prime 2.0 software (Psychological Software Tools, Inc., Pittsburgh, PA, United States) and were projected onto the screen.

### fMRI Data Analysis

Imaging data analysis, including pre-processing, was performed using Statistical Parametric Mapping 5 (SPM5) software (Wellcome Department of Imaging Neuroscience, London, United Kingdom) in Matlab 7.0 (Mathworks Inc., United States). The data were acquired and analyzed sequentially as follows. During the preprocessing stage, functional images were first realigned to the first volume and corrected for head motion. Since no participant exceeded the maximum head motion of 3 mm in any direction, we performed slice timing with all participants’ imaging data after the realignment. The data were then normalized to the standard space defined by the Montreal Neurological Institute echo-planar imaging (EPI) template and were converted to Talairach using Brett’s mni2tal method. After normalization, the data were spatially smoothed to 8 mm using a full-width-at-half-maximum isotropic (FWHM) Gaussian kernel.

After preprocessing, the data from each participant were modeled and statistically analyzed using the general linear model (GLM) approach as implemented in SPM5 software. One-sample *t*-tests were conducted to compare the differential brain activations depending on the different social contextual effects. Three regressors (pain stimuli onset time, neutral stimuli onset time, and task response time), one parametric modulator (correctness of each trial), and six motion parameters for each version of the task were included in the final model. The correctness parametric modulator was included to control for unintended neural responses caused by different levels of accuracy between the social contexts.

Planned contrasts were constructed for the general linear models. A series of planned contrasts for the whole-brain group level *t*-tests and conjunction analyses were conducted. To examine the first research question regarding the social contextual effects on the cognitive processes while participants were solving the spatial perception task, we conducted whole-brain group level *t*-tests with the following four contrasts. (1) The task phase under cooperation (cooperation_task) > the task phase under the individual context (individual_task); (2) the task phase under competition (competition_task) > (individual_task); (3) (cooperation_task > competition_task); and (4) (competition_task > cooperation_task). In addition, we also conducted a conjunction analysis with contrasts of (cooperation_task > individual_task) and (competition_task > individual_task) to examine the commonly activated brain regions between cooperation and competition conditions during the task phase in comparison with the baseline condition.

To test the second research question about the social contextual effects on the empathic neural responses when the participants were exposed to empathy-evoking stimuli, we conducted whole-brain group level *t*-tests with the following four contrasts. (1) The painful stimuli presentation under cooperation (cooperation_pain) > the painful stimuli presentation under individual context (individual_pain); (2) the painful stimuli presentation under competition (competition_pain) > (individual_pain); (3) (cooperation_pain > competition_pain); and (4) (competition_pain > cooperation_pain). We also conducted a conjunction analysis with contrasts of (cooperation_pain > individual_pain) and (competition_pain > individual_pain) to examine the commonly activated brain regions between cooperation and competition conditions during the stimuli phase in comparison with the baseline condition.

To explore the third research question regarding the modulation of trait empathy in the contextual effects on the state empathy, we conducted correlation analyses between trait empathy and state empathic neural responses in *a priori* brain regions including the AIC and dACC known as an empathy network. We extracted the average neural activity of the ROIs for each social context using the Marsbar toolbox (Brett et al., 2002).

In general, results at a threshold of *p* < 0.001 uncorrected and of more than 10 voxels were considered. For supplementary information, we reported statistical strengths of neural responses at a marginally significant voxel-wise level of *p* ≤ 0.06 false discovery rate (FDR) corrected for multiple comparisons. Activations in *a priori* ROIs which survived in the whole-brain correction were subject to small-volume correction (SVC). ROI masks for SVC were created based on *a priori* anatomical structures found in previous empirical studies. Masks for the dmPFC and vmPFC were created as cooperation-relevant brain regions based on previous studies (Decety et al., 2004; Elliott et al., 2006). More precisely, the masks for the bilateral dmPFC were created as 10-mm spheres centered on the coordinates (*x* = 3, *y* = 51, *z* = 25; *x* = -6, *y* = 51, *z* = 28) identified in an Elliott et al.’s (2006) empirical study. The mask for the left vmPFC was also created as a 10-mm sphere centered on the coordinates (*x* = -12, *y* = 31, *z* = -10) reported in a Decety et al.’s (2004) study. Masks for the AIC and dACC were also created as the representative empathy network in the human brain. Spheres 10 mm in size centered on the left AIC (*x* = -40, *y* = 10, *z* = 0) and the left dACC (*x* = -9, *y* = 19, *z* = 40) were created based on a Jackson et al.’s (2005) study. These regions are closely associated with empathy for others’ pain. Moreover, because we adopted the same empathy stimuli as Jackson et al. (2005), the coordinates of the empathy network were highly likely to appear in our data set.

## Results

### Behavioral Results

**FIGURE 2:**
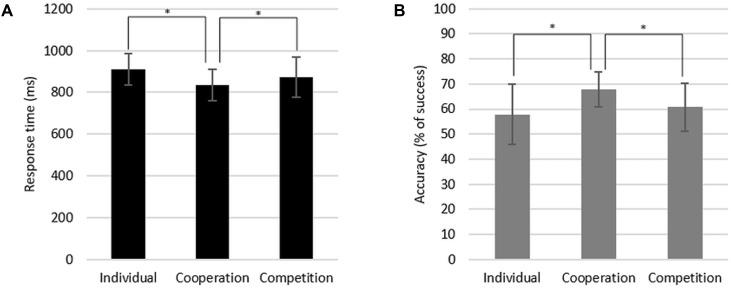
Response time **(A)** and accuracy **(B)** of the task performance during the individual, cooperation, and competition conditions.

We conducted two repeated measures analyses of variance (ANOVAs) to test the differences in participants’ response time and accuracy during the task according to the social context. There were significant differences in both response time [*F*_(2,14)_ = 9.64, *p* < 0.01] and accuracy [*F*_(2,14)_ = 8.98, *p* < 0.01] depending on the context (**Figure 2**). We then conducted paired sample *t*-tests as *post hoc* analyses. Participants responded faster under cooperation (*M* = 833.80 ms, *SD* = 75.66 ms) than under competition [*M* = 873.69 ms, *SD* = 96.69 ms, *t*_(15)_ = -3.11, *p* < 0.01] and the individual context [*M* = 909.27 ms, *SD* = 74.50 ms, *t*_(15)_ = -4.13, *p* < 0.01; **Figure 2A**]. A similar pattern was also found in accuracy score. Participants performed the task more accurately for the cooperative version of the task (*M* = 67.75%, *SD* = 6.97%) than for the competitive version [*M* = 60.78%, *SD* = 9.69%, *t*_(15)_ = 2.84, *p* < 0.05] and for the individual version [*M* = 57.88%, *SD* = 12.03%, *t*_(15)_ = 4.58, *p* < 0.01; **Figure 2B**]. There was no statistically significant difference between the individual and competition conditions in both response time and accuracy. These differences between cooperation and competition conditions imply that participants showed the significantly higher accuracy rate and faster response time when performing task under the cooperative context compared to under the competitive context. We did not find any statistical differences in empathy ratings for painful stimuli between the different social contexts.

### fMRI Results

#### Effects of Social Contexts on Social Cognitive Brain Activations During the Task Phase

First, two general linear models of (cooperation_task > individual_task) and (competition_task > individual_task) were tested in order to explore the first research question on the distinctive neural responses to the cooperative and competitive social contexts in comparison with the individual context as a baseline when participants solved the spatial perception task. **Table 1** reports distinctive brain activations under the cooperative and competitive contexts during the task phase, respectively. The results supported our hypotheses that the cooperative social context activated brain regions related to mentalizing and spatial cognitive processing, whereas the competitive social context activated them weakly. The (cooperation_task > individual_task) contrast showed neural responses in brain regions related to mentalizing, such as the bilateral dmPFC, temporal pole, and left TPJ. In addition, the contrast also reported brain activations in the bilateral caudate nucleus, dlPFC, vlPFC, left superior parietal lobe, and left AG, which are relevant to spatial perception and mental rotation. All activations were found to be at uncorrected *p* < 0.001 uncorrected (FDR corrected at *p* = 0.06) with the minimal cluster size *k* is 10 voxels. The (competition_task > individual_task) contrast also showed brain activations in the bilateral dlPFC, caudate nucleus, and AG. However, the contrast showed relatively weak activations in mentalizing regions compared to the (cooperation_task > individual_task) contrast, based on the activations only in the left superior temporal gyrus at *p* < 0.001 uncorrected, extent threshold *k* > 10.

**Table 1:** Brain activations in (Cooperation_Task > Individual_Task) and (Competition_Task > Individual_Task) contrasts.

Region	BA	R/L	Talairach coordinate			Voxel (*k*)	*z-*value
			*x*	*y*	*z*		
**Cooperation_Task > Individual_Task**							
Dorsomedial prefrontal cortex^∗^	32	R	13	32	19	914	4.67
	8	L	-7	23	51	54	3.62
Thalamus^∗^	50	L	-13	-13	8	156	4.59
	50	R	9	-11	3	19	3.49
Caudate nucleus^∗^	48	L	-6	11	2	291	4.56
	48	R	9	11	0	37	3.67
Dorsolateral prefrontal cortex^∗^	8	R	22	24	43	243	4.50
	8	L	-41	10	34	201	3.99
	9	R	27	39	25	48	3.73
Temporal pole^∗^	38	L	-36	8	-23	155	4.38
	38	R	27	6	-24	79	3.61
Posterior cingulate cortex^∗^	23	L	-5	-48	21	107	4.00
Ventrolateral prefrontal cortex^∗^	44	R	58	20	25	52	3.89
	47	R	47	22	-2	16	3.55
	45	L	-54	19	22	22	3.49
Temporoparietal junction^∗^	39	L	-51	-49	20	43	3.50
Primary visual cortex^∗^	17	R	16	-81	11	11	3.40
Superior parietal lobe^∗^	19	L	-20	-63	27	19	3.30
Angular gyrus^∗^	39	L	-56	-60	30	10	3.30
**Competition_Task > Individual_Task**							
Dorsolateral prefrontal cortex	9	L	-33	27	25	231	4.62
	8	R	24	18	39	32	4.27
	9	R	27	38	25	233	4.13
Premotor cortex	6	R	18	20	55	91	3.97
Primary sensory cortex	1	R	48	-15	48	33	3.94
Angular gyrus	39	L	-60	-47	21	18	3.85
Superior temporal gyrus	22	L	49	-47	7	28	3.62
Thalamus	50	R	9	-13	13	17	3.60
Caudate nucleus	48	R	17	11	3	42	3.58
	48	L	-13	8	13	11	3.47
Primary visual cortex	17	R	16	-75	9	23	3.57
Lingual gyrus	19	L	-15	-48	0	52	3.53
	19	R	25	-50	2	64	3.38

*^∗^p = 0.06 FDR corrected, extent threshold k > 10. p < 0.001 uncorrected, extent threshold k > 10.*

**FIGURE 3:**
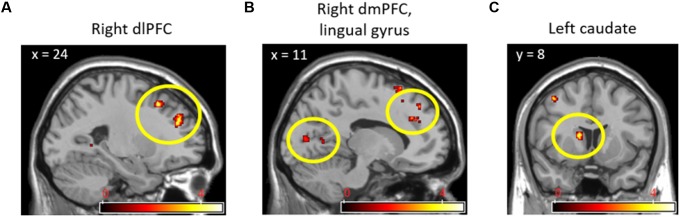
Right dlPFC **(A)**, right dmPFC and lingual gyrus **(B)**, and left caudate nucleus **(C)** activations from conjunction analyses with (Cooperation_Task > Individual_Task) and (Competition_Task > Individual_Task) contrasts.

While observing the commonalities in the neural responses between the two social contexts, we conducted a conjunction analysis in order to explore the extent to which brain regions were commonly activated by social contexts compared to the individual context. **Table 2** displays the results from a conjunction analysis investigating common brain activations between (cooperation_task > individual_task) and (competition_task > individual_task) contrasts. The bilateral dlPFC (**Figure 3A**), right dmPFC and lingual gyrus (**Figure 3B**), and left caudate nucleus (**Figure 3C**) were commonly activated by two different social contexts at *p* < 0.001 uncorrected, extent threshold *k* > 10. That is, both social contexts heightened brain activations associated with monitoring not only one’s own cognitive processing, but also other’s by reading other’s mind.

**Table 2:** Brain activations from conjunction analysis with (Cooperation_Task > Individual_Task) and (Competition_Task > Individual_Task) contrasts.

Region	BA	R/L	Talairach coordinate			Voxel (*k*)	*z-*value
			*x*	*y*	*z*		
Dorsolateral prefrontal cortex	6	R	20	20	53	77	4.12
	9	R	27	39	25	83	4.09
	8	R	24	20	43	34	3.97
	9	L	-31	26	35	32	3.83
	6	L	-41	10	46	14	3.41
Primary visual cortex	17	R	16	-75	9	28	3.66
Caudate nucleus	48	L	-13	8	13	26	3.66
Thalamus	50	L	-13	-13	8	14	3.54
Dorsomedial prefrontal cortex	9	R	11	33	28	18	3.41
	8	R	11	39	40	11	3.31
Lingual gyrus	19	R	20	-53	2	12	3.20

*p < 0.001 uncorrected, extent threshold k > 10.*

**FIGURE 4:**
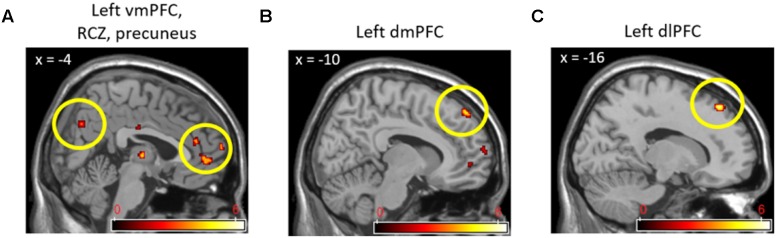
Left vmPFC, RCZ, precuneus **(A)**, left dmPFC **(B)**, and left dlPFC **(C)** activations in (Cooperation_Task > Competition_Task) contrast.

In direct comparisons between the cooperative and competitive conditions of the experiment, only the (cooperation_task > competition_task) contrast produced statistically significant neural activations. **Table 3** and **Figure 4** show the brain activations in the left vmPFC, left rostral cingulate zone (RCZ), left precuneus (**Figure 4A**), bilateral dmPFC (**Figure 4B**), left dlPFC (**Figure 4C**), and left thalamus. Activations in the left vmPFC (*p* < 0.05 small-volume FDR corrected, *k* = 22) and bilateral dmPFC (*p* < 0.05, small-volume FDR corrected, *k* = 11) were also found from small-volume correction analysis with *a priori* defined anatomical ROIs. Despite the marginal statistical significance, the left globus pallidus activation (*x* = -21, *y* = -10, *z* = -5; *p* < 0.005 uncorrected, *k* = 15 voxels) was also found in this contrast. In the (competition_task > cooperation_task) contrast, the cerebellum (*x* = 0, *y* = -50, *z* = 0; 46 voxels) and the right AIC (*x* = 33, *y* = 33, *z* = 4; 15 voxels) were marginally activated at *p* < 0.005 uncorrected.

**Table 3:** Brain activations in (Cooperation_Task > Competition_Task) contrast.

Region	BA	R/L	Talairach coordinate			Voxel (*k*)	*z-*value
			*x*	*y*	*z*		
Ventromedial prefrontal cortex^∗^	10	L	-4	61	7	22	3.71
	10	L	-4	46	-3	61	3.40
Dorsomedial prefrontal cortex^∗^	8	L	-10	47	43	26	3.72
	9	R	7	48	28	13	3.31
Rostral cingulate zone	32	L	-2	37	14	11	3.43
Precuneus	7	L	-4	-74	34	19	3.37
Dorsolateral prefrontal cortex	8	L	-16	40	47	41	4.38
Thalamus	50	L	-1	-13	6	22	3.64

*^∗^p < 0.05 FDR small volume corrected, extent threshold k > 10. p < 0.001 uncorrected, extent threshold k > 10.*

As a supplementary analysis, we conducted a separate regression analysis on brain activation during the task phase with an individual’s average accuracy score for each version of the task. Under the cooperative context, accuracy score was positively related to neural activations in the right TPJ (*x* = 49, *y* = -34, *z* = 29; 30 voxels), right retrosplenial cortex (RSC; *x* = 12, *y* = -42, *z* = 2; 30 voxels), and right precuneus (*x* = 16, *y* = -49, *z* = 37; 15 voxels) at *p* < 0.001 uncorrected. Under the competitive context, however, accuracy score was positively related to the neural activations in the left posterior cingulate cortex (*x* = -5, *y* = -22, *z* = 42; 57 voxels) and left supplementary motor cortex (SMA; *x* = -1, *y* = -3, *z* = 51; 22 voxels) at *p* < 0.001 uncorrected.

#### Effects of Social Contexts on Empathic Brain Activations During the Pain Stimuli Phase

Two contrasts, (cooperation_pain > individual_pain) and (competition_pain > individual_pain), were applied to explore the second research question on the differential neural activations in response to other’s pain under the different social contexts (cooperation and competition) compared to the individual context as a baseline. **Table 4** presents distinctive brain activations under the cooperation and competition contexts during the pain stimuli phase, respectively. The results partially supported our hypotheses that the cooperative context activated brain regions related to empathy network than the individual context, whereas the competitive context activated them weakly. The cooperative context activated neural responses related to empathic responses, including the right insular cortex, left dACC, left STS, and left SMA than the individual context at *p* < 0.05 FDR corrected, extent threshold *k* > 10. The competitive context also activated the right posterior insular cortex, pMCC, and the dmPFC associated with the empathy network compared with the individual context at *p* = 0.001 uncorrected (*p* = 0.06 FDR corrected), extent threshold *k* > 10.

**Table 4:** Brain activations in (Cooperation_Pain > Individual_Pain) and (Competition_Pain > Individual_Pain) contrasts.

Region	BA	R/L	Talairach coordinate			Voxel (*k*)	*z-*value
			*x*	*y*	*z*		
**Cooperation_Pain > Individual_Pain**							
Caudate nucleus^∗^	48	R	17	17	0	3653	5.24
Superior temporal sulcus^∗^	22	L	-49	-32	16	487	4.77
Posterior insular cortex^∗^	13	R	33	-19	9	273	4.58
	13	R	36	-14	-4	36	3.73
Angular gyrus^∗^	39	R	39	-55	34	1485	4.38
	39	L	-42	-61	36	1376	3.90
Dorsal posterior cingulate cortex^∗^	31	L	-7	-38	31	3293	4.33
Superior parietal lobe^∗^	7	L	-14	-40	64	60	3.83
Dorsolateral prefrontal cortex^∗^	9	L	-58	9	27	61	3.71
	8	R	41	22	40	46	3.25
Posterior midcingulate cortex^∗^	31	R	12	-12	42	33	3.67
Dorsal entorhinal cortex^∗^	34	L	-14	8	-13	182	3.64
Supplementary motor area^∗^	6	L	-1	-22	58	134	3.52
Anterior insular cortex^∗^	13	R	34	11	3	10	3.42
Primary sensory cortex^∗^	1	R	29	-26	51	150	3.38
Cerebellum^∗^			0	-61	-11	98	3.35
Parahippocampal gyrus^∗^	36	L	-21	-21	-13	14	3.13
	34	R	17	2	-10	13	2.93
Primary motor cortex^∗^	4	L	-40	-10	20	15	3.12
Ventrolateral prefrontal cortex^∗^	44	R	55	11	22	28	3.07
Premotor cortex^∗^	6	R	22	-7	44	23	3.02
Dorsal anterior cingulate cortex^∗^	24	L	-7	-3	46	11	2.97
**Competition_Pain > Individual_Pain**							
Angular gyrus	39	R	47	-66	39	202	4.64
Posterior midcingulate cortex	31	R	12	-12	41	81	4.30
Dorsal posterior cingulate cortex	23	R	6	-53	12	228	4.29
Hippocampus	54	R	32	-21	-6	55	4.13
Dorsomedial prefrontal cortex	9	R	3	43	18	94	4.10
Supramarginal gyrus	40	R	52	-25	19	216	3.98
Cerebellum		R	18	-54	-11	109	3.91
Primary motor cortex	4	R	25	-30	53	88	3.83
	4	L	-16	-30	64	31	3.81
Posterior insular cortex	13	R	31	-16	19	96	3.82
	13	L	-38	-16	-1	10	3.43
Caudate nucleus	48	R	9	0	18	26	3.81
Primary sensory cortex	1	R	42	-21	41	83	3.78
Dorsolateral prefrontal cortex	10	R	39	38	22	13	3.25

*^∗^p < 0.05 FDR corrected, extent threshold k > 10. p < 0.001 uncorrected, extent threshold k > 10 (p = 0.06 FDR corrected).*

**FIGURE 5:**
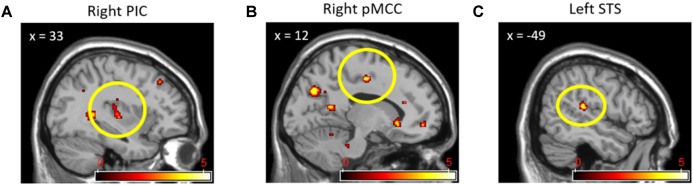
Right posterior insular cortex **(A)**, right pMCC **(B)**, and left STS **(C)** activations from conjunction analyses with (Cooperation_Pain > Individual_Pain) and (Competition_Pain > Individual_Pain) contrasts.

We also conducted a conjunction analysis in order to explore which brain regions were commonly activated by social contexts compared to the individual context while participants were witnessing other’s pain. **Table 5** displays the results from the conjunction analysis investigating shared brain activations between (cooperation_pain > individual_pain) and (competition_pain > individual_pain) contrasts. Particularly, the right posterior insular cortex (**Figure 5A**), right pMCC (**Figure 5B**), and left STS (**Figure 5C**), related to empathy network, were commonly activated by two social contexts at *p* < 0.001 uncorrected, extent threshold *k* > 10.

**Table 5:** Brain activations from conjunction analysis with (Cooperation_Pain > Individual_Pain) and (Competition_Pain > Individual_Pain) contrasts.

Region	BA	R/L	Talairach coordinate			Voxel (*k*)	*z-*value
			*x*	*y*	*z*		
Angular gyrus	39	R	45	-66	41	273	4.41
Posterior insular cortex	13	R	33	-19	9	89	4.39
Caudate nucleus	48	R	17	17	0	86	4.17
Posterior midcingulate cortex	24	R	12	-12	41	24	4.12
Superior temporal sulcus	22	L	-49	-32	16	42	4.02
	40	R	50	-25	19	52	3.77
Posterior cingulate cortex	23	R	10	-51	14	37	3.75
	31	L	-9	-40	32	74	3.59
Ventromedial prefrontal cortex	11	L	-18	43	-8	14	3.60
	10	R	13	44	-8	16	3.43
Dorsolateral prefrontal cortex	8	R	36	32	40	17	3.58
	9	R	41	40	24	25	3.47
Primary motor cortex	4	R	25	-30	56	12	3.30

*p < 0.001 uncorrected, extent threshold k > 10.*

**FIGURE 6:**
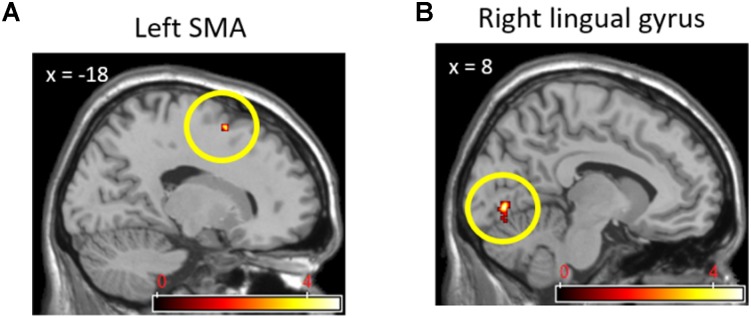
Left SMA **(A)** activation in (Cooperation_Pain > Competition_Pain) contrast and right lingual gyrus **(B)** activation in (Competition_Pain > Cooperation_Pain) contrast.

Direct comparisons between cooperation and competition during the pain stimuli phase produced activations in only a few brain regions that we did not expect. In the (cooperation_pain > competition_pain) contrast, activation of the left SMA (*x* = -18, *y* = 9, *z* = 53; 10 voxels; **Figure 6A**) was found, whereas in the (competition_pain > cooperation_pain) contrast, the right lingual gyrus (*x* = 8, *y* = -76, *z* = 1; 33 voxels; **Figure 6B**) was found to be activated at *p* < 0.001 uncorrected. In order to interpret these findings, we conducted a regression analysis on brain activation during the pain stimuli phase with the pain ratings for each social context. We found that, under competition, activations in the left lingual gyrus (*x* = -23, *y* = -46, *z* = -2; 15 voxels), right pMCC (*x* = 10, *y* = -19, *z* = 44; 17 voxels), and right primary motor cortex (*x* = 31, *y* = -27, *z* = 48; 17 voxels) were positively correlated with the pain ratings at *p* < 0.001 uncorrected. However, under cooperation, only the left amygdala (*x* = -18, *y* = -8, *z* = -6; 24 voxels) was marginally correlated with the pain ratings at *p* < 0.005 uncorrected.

#### Interaction Between Trait- and State-Empathy Under Different Social Contexts

**FIGURE 7:**
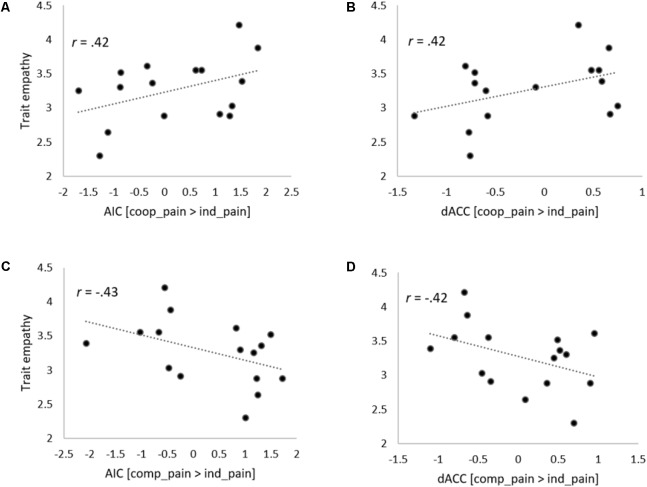
Scatter plots describing correlations between individual’s trait empathy score and state empathic neural responses. Correlations between trait empathy and signal changes in the AIC **(A)** and dACC **(B)** activations in (Cooperation_Pain > Individual_Pain) contrast. Correlations between trait empathy and signal changes in the AIC **(C)** and dACC **(D)** activations in (Competition_Pain > Individual_Pain) contrast.

The ROIs and correlation analyses were conducted to examine the third research question regarding the modulation effect of trait empathy on the neural activity in independent brain regions, which were found in previous research to be closely related to the current study. Correlation coefficients between the trait empathy score and the average neural activities of ROIs from each contrast were analyzed. As a result, we found that our hypotheses about the modulation of trait empathy in different contextual effects on neural responses were partially supported in the empathic brain activations during the pain stimuli phase, but not in the brain activations during the task phase. More precisely, the correlations between the trait empathy and the average neural activities in the left AIC and left dACC for the (cooperation_pain > individual_pain) contrast (*r*s = 0.42, 0.42, *p*s = 0.05, 0.05 uncorrected) were significantly different from those for the (competition_pain > individual_pain) contrast (*r*s = -0.43, -0.42, *p*s = 0.05, 0.05 uncorrected) from the Fisher’s *Z*-tests (*Z*s = 2.30, 2.27, *p*s = 0.02, 0.02). **Figure 7** shows that these correlations were not resulted from outliers though, we need to inform that the statistical power of each correlation coefficient was not strong due to the small sample size. The correlation coefficients between the trait empathy and the average neural activities in the left vmPFC and bilateral dmPFC for the (cooperation_task > individual_task) contrast (*r*s = 0.26, 0.02, 0.20, *p*s = 0.16, 0.47, 0.23 uncorrected) did not show statistically significant differences from those of the (competition_task > individual_task) contrast (*r*s = -0.40, -0.21, -0.09, *p*s = 0.06, 0.22, 0.38 uncorrected) based on the Fisher’s *Z*-test (*Z*s = 1.77, 0.59, 0.74, *p*s = 0.07, 0.56, 0.46).

## Discussion

We investigated the underlying mechanisms of cooperation and competition in relation to neural responses by having participants help or disrupt another player during a Tetris-like game. The results confirmed our hypotheses that, when individuals completed the task in a cooperative context, they performed more quickly and accurately and demonstrated stronger neural activation in the mentalizing network and the reward-related brain regions than what they did when in the individual or competitive context. The effects of cooperation on the social cognitive neural responses were stronger for individuals with higher trait empathy scores than those with lower scores. More empathic individuals exhibited stronger empathic responses toward the pain of others in a cooperative context. These behavioral and neural findings are consistent with previous behavioral and fMRI studies on the effects of cooperation and competition.

### Cooperative and Competitive Contextual Effects on Social Cognitive Processes

The first research hypothesis on the shared but distinctive effects of cooperation and competition on social cognitive processing was supported by the current findings. First of all, in comparison with individual task performance (i.e., the absence of contextual cues of social interaction), both the cooperative and competitive contexts activated mentalizing-related regions, such as the bilateral dmPFC. The activation in mentalizing regions is consistent with findings from earlier studies on cooperation and competition (Rilling et al., 2002; Decety et al., 2004; Halko et al., 2009). In addition to the heightened activity in the dmPFC area, increased activations in the dlPFC further support the idea that social context requires executive functioning and cognitive inference skills in order to read other people’s minds (Alvarez and Emory, 2006). The stronger spatial cognitive processing, which was the target task of the present study, was also found based on the activations in the primary visual cortex, lingual gyrus, and AG during the spatial perception task performance under the social contexts compared to the individual context (Seghier, 2013). This interpretation on the common social cognitive mechanism underlying two representative social contexts was also supported by the conjunction analysis. To synthesize, both cooperative and competitive contexts result in people’s intention to read other people’s mind, which in turn leads to more active task engagement.

The cooperative social context also showed activations in the unique neural system compared to the competitive social context. More precisely, stronger activation in the left vmPFC under cooperation than competition was found, which was consistent with previous findings of fMRI studies that treated cooperation as a reciprocal and direct social interaction with other people (McCabe et al., 2001; Rilling et al., 2002; Decety et al., 2004; King-Casas et al., 2005; Stallen and Sanfey, 2013). According to them, cooperation itself is psychologically rewarding for human beings (Tabibnia and Lieberman, 2007). Similar activation in the vmPFC was also found when participants were asked to donate money to the needy (Moll et al., 2006; Harbaugh et al., 2007; Hare et al., 2010) or to infer others’ intentions to donate (Cooper et al., 2010). These findings can be extended to our own in that our participants were instructed to “help” the other player. It can be concluded that the intention to help others can be socially rewarding in itself. Marginally significant activation in the globus pallidus (15 voxels; *p* < 0.005, uncorrected), which is known to be an important component of the reward system, also supports the interpretation that activation in the vmPFC is related to the reward process in a cooperative context. In other words, our participants were intrinsically satisfied while helping the other person even without positive feedback or direct interaction with other people.

In addition to the vmPFC, other prefrontal regions demonstrated greater activation in the cooperative social context than in the competitive one. The bilateral dmPFC, the left RCZ, and the left dlPFC are closely related to the executive function of social cognition. The dmPFC and RCZ are known to be regions that govern social cognitive mentalizing in human brain (Amodio and Frith, 2006; Lieberman, 2007). The dlPFC, which may not have a direct influence on mentalizing *per se*, could contribute to executive functioning and strong cognitive engagement during the task (Ozonoff et al., 1991; Cole and Mitchell, 2000; Carlson et al., 2002; Abe et al., 2007; Polosan et al., 2011). In other words, both social and cognitive processes were more active when the task was performed in the cooperative context than in the competitive context.

Nevertheless, the greater activation in the vmPFC during cooperation might have been resulted by the internal satisfaction with the better performance in the cooperation condition than in the competition condition. In order to prevent the potential confounding effect of task accuracy, we included correctness of each trial as a parametric modulator in our general linear models and controlled them in all of the present analyses. Furthermore, according to our previous pilot results, there was no statistical difference in participants’ perception of task difficulty between the cooperative and competitive tasks (*M*_cooperation_ = 3.59, *SD*_cooperation_ = 1.44; *M*_competition_ = 3.82, *SD*_competition_ = 1.22; *t*_20_ = -0.64, *p* = 0.31). Thus, it is implausible to suppose that participants were aware of their differential accuracy rate depending on the social contexts during scanning.

We did not find any significantly stronger neural responses in the presence of competition. This could be related to the lower accuracy and slower response time that the participants showed when they solved the competitive version of the task. The competitive context marginally activated the right AIC and cerebellum, known as the psychological pain matrix (Panksepp, 2003; Jackson et al., 2006; Schraa-Tam et al., 2011), despite the overall weak neural activations. According to previous studies, the AIC tends to be activated when people witness unfairness or deception during social interaction (Lissek et al., 2008; Rilling and Sanfey, 2011). The uncomfortable feelings provoked by the fact that they had to interfere with their opponent’s task performance might have hindered cognitive engagement and behavioral readiness (Herrington et al., 2005). Based on this, we can infer that the negative emotions generated by the competitive context might have suppressed the participants’ social and cognitive processes, which were required for successful task performance.

In addition, the correlation between task accuracy scores and neural activation for each social context supported our interpretation about the whole brain analysis. The more accurate scores participants obtained while completing the cooperative version of the task, the stronger activation in the mentalizing regions, including the right TPJ and precuneus was found. Particularly, activation in the RSC can be interpreted as the linkage between egocentric and allocentric spatial processing, which was essentially required in the current Tetris-like game (Byrne et al., 2007; Epstein, 2008; Vann et al., 2009). That is, under cooperation, individuals who were better at the task demonstrated better compatibility between egocentric and allocentric spatial processing and better mentalizing other’s mind. In contrast, for the competitive version of the task, the higher the task accuracy participants had, the stronger the activation in the left SMA and mid cingulate cortex was found. In other words, when participants were asked to help the other person, they thought more carefully about what the other person was thinking, which in turn led to a better task accuracy. However, when they were asked to interrupt the competitor, their mentalizing system did not seem to function in an adaptive way to produce better performance.

### Cooperative and Competitive Contextual Effects on Empathic Process

The second research hypothesis on the contextual effects on emotional empathic process was partially supported by the current finding. Above all, regardless of the social contextual types, participants showed increased empathic neural activations in response to other’s pain in both cooperative and competitive contexts compared to the individual context as a baseline. The simple awareness of social interactions without actual reciprocal interaction can make individual more sensitive to other people’s intention and emotion, which in turn might have led to the increased empathic responses. From this finding, we could confirm the effects of contextual cues related to cooperation and competition on not only social cognitive, but also emotional responses.

However, even though we had expected that more empathy-related brain regions would be activated during the cooperative version of the task compared to the competitive version when participants saw another person in pain, we did not find any direct evidence of this from the whole brain analysis. This could have been due to the modulation of individual differences. Feeling empathic toward others’ pain can be modulated by individual differences in the sensitivity toward others’ pain, i.e., trait empathy. According to previous research about the neural mechanisms for empathy, empathic neural responses toward empathy-evoking stimuli tend to be modulated by the perceived salience of the stimuli (for a review, see Jackson et al., 2005; Lamm et al., 2011). That is, how strongly and saliently the pain of others is perceived at any particular moment may modulate empathic neural responses. In a similar vein, we found a marginal correlation between activation in the amygdala and pain ratings while participants were helping the opposite player during the cooperative version of the task. In other words, when participants felt more empathic toward the person experiencing pain during the cooperation task, brain activation related to negative emotions would have become stronger (Moll et al., 2002; Carr et al., 2003; Völlm et al., 2006). Even so, more evidence is required to support our hypothesis about contextual effects on emotional processes.

### Modulation of Trait Empathy in the Contextual Effects on State Empathy

In support of previous research on interaction between trait- and state-empathy, we found that the relationship between trait empathy scores and empathic neural responses might vary depending on the social contexts. When empathy-evoking stimuli were presented in the cooperative context, more empathic individuals tended to show stronger activations in the AIC and dACC, which are closely associated with empathy for others’ pain, than in the individual context. On the contrary, there were patterns of negative relationship between trait empathy and activations in the AIC and dACC under the competitive context in spite of the statistical non-significance. This finding can be carefully interpreted that individuals with high trait empathy might become easily immersed in the cooperation and felt more attached to the cooperator. Consequently, this increased attachment to the cooperator might have led empathic individuals to feel greater empathy for others’ pain. In contrast, the empathic individuals might have felt uncomfortable to get involved in the competitive context where their natural empathic responses were inhibited due to the potential incompatibility with their personality trait (Gilin et al., 2013). Otherwise, empathic individuals might have reacted more exclusively toward the opponent, who was regarded as the out-group member, under the competitive context (Azevedo et al., 2013). Trait empathy might be revealed only for in-group members, but not for out-group members or opponents. However, the neural activities in the vmPFC and dmPFC did not seem to be modulated by trait empathy during task performance. This could be because the current measurement of trait empathy represents emotional aspect of empathy rather than cognitive perspective taking (Mehrabian and Epstein, 1972). Throughout these results, we carefully conclude that individual’s trait empathy modulated state empathic emotional responses toward other’s pain. Still, since the statistical power of each correlation between trait- and state-empathy was not strong, further investigations need to address this issue in a larger sample.

### Limitations and Future Directions

Despite the aforementioned unique contribution of the current study, we need to mention the low statistical power caused by the small sample size and no jittering as a caveat of this study. Although sixteen participants could not be enough to make generalization of the current findings, we believe that the present data provided useful information regarding the effect of cooperation versus competition on social cognitive and empathic neural responses. As a partial compensation for the small sample size, our fMRI findings showed stable brain activations even after correcting the increased type 1 errors of multiple comparisons. To synthesize, we could carefully claim that the current findings might not result from the false positives caused by small sample size, but replication in the larger sample needs to be addressed in the future research. The other potential limitation is the non-jittered experimental design, which might be critical to given how slow the hemodynamic response is. Nevertheless, with randomly displayed empathy-evoking pain stimuli, we have attempted to minimize this potential drawback. Random appearance of pain stimuli during the task performance could function as a quasi-jitter during the scanning. Although brain activations associated with pain stimuli might include “leaked” activations from task events, all conditions contained the potential “leaked” activations regardless of the social contexts (Zhang et al., 2012; Yang et al., 2013). In addition, the ratio between the presence of painful images and neutral images was identical across conditions to control the emotional valence that elicited. Thus, we carefully argue that the potential issue regarding the jittering might have influences on the BOLD signals, but its influences were equivalent across the condition. These common effects of non-jittering might be canceled out by our GLMs embracing between-condition contrasts.

## Conclusion

From our findings, it can be concluded that cooperation leads to more adaptive behavioral, cognitive, and emotional responses, especially for highly empathic individuals. A cooperative context based on a simple behavioral goal – helping another person without reciprocal interaction or feedback – was sufficient to provoke more desirable behavioral and neural responses. In a cooperative context, participants performed better, felt more satisfied with their helping behavior, and mentalized the opposing player better than what they did when in the competitive context. Furthermore, empathic individuals empathized more with others’ pain in a cooperative context than in a competitive context.

## Author Contributions

All authors listed have made a substantial, direct and intellectual contribution to the work, and approved it for publication.

## Conflict of Interest Statement

The authors declare that the research was conducted in the absence of any commercial or financial relationships that could be construed as a potential conflict of interest.

## Footnotes

1 We recruited only male participants to control for potential sex differences in two aspects. First, there could be a sex difference in perceived competitiveness during the competitive task, which might cause different patterns of neural activations (for a review, see Niederle and Vesterlund, 2011). Second, there also exists a sex difference in performance of the spatial ability task (Linn and Petersen, 1985; Voyer et al., 1995), which might be rooted in the different activities of brain regions underlying spatial cognition such as inferior parietal lobe (Thomsen et al., 2000; Weiss et al., 2003; Schöning et al., 2007). Our pilot study using the identical task with 22 college students (11 males) confirmed that the sex difference existed in task performance, *t*_20_ = 2.12, *p* = 0.047 (*M*_male_ = 76.70%, *SD*_male_ = 7.38; *M*_female_ = 67.06%, *SD*_female_ = 13.17).
